# Simple Nutrient-Based Rules vs. a Nutritionally Rich Plant-Centered Diet in Prediction of Future Coronary Heart Disease and Stroke: Prospective Observational Study in the US

**DOI:** 10.3390/nu14030469

**Published:** 2022-01-21

**Authors:** Yuni Choi, Daniel D. Gallaher, Karianne Svendsen, Katie A. Meyer, Lyn M. Steffen, Pamela J. Schreiner, James M. Shikany, Jamal S. Rana, Daniel A. Duprez, David R. Jacobs

**Affiliations:** 1 Division of Epidemiology and Community Health, University of Minnesota–Twin Cities, Minneapolis, MN 55454, USA; choi0518@umn.edu (Y.C.); steff025@umn.edu (L.M.S.); schre012@umn.edu (P.J.S.); 2 Department of Food Science and Nutrition, University of Minnesota–Twin Cities, Saint Paul, MN 55108, USA; dgallahe@umn.edu; 3 Department of Nutrition, University of Oslo, 0317 Oslo, Norway; karianne.svendsen@medisin.uio.no; 4 The Lipid Clinic, Department of Endocrinology, Morbid Obesity and Preventive Medicine, Oslo University Hospital, 0424 Oslo, Norway; 5 Department of Nutrition and Nutrition Research Institute, University of North Carolina—Chapel Hill, Chapel Hill, NC 28081, USA; ktmeyer@email.unc.edu; 6 Division of Preventive Medicine, School of Medicine, University of Alabama at Birmingham, Birmingham, AL 35205, USA; jshikany@uabmc.edu; 7 Divisions of Cardiology and Research, Kaiser Permanente Northern California, Department of Medicine, University of California, Oakland, CA 94612, USA; jamal.s.rana@kp.org; 8 Cardiovascular Division, Department of Medicine, University of Minnesota–Twin Cities, Minneapolis, MN 55454, USA

**Keywords:** coronary heart disease, cholesterol-lowering diets, simple nutrient-based rules, plant-centered diets, stroke, prospective study

## Abstract

To better understand nutrition paradigm shift from nutrients to foods and dietary patterns, we compared associations of a nutrient-based blood cholesterol-lowering diet vs. a food-based plant-centered diet with risk of coronary heart disease (CHD) and stroke. Participants were 4701 adults aged 18–30 years and free of cardiovascular disease at baseline, followed for clinical events from 1985 and 86 to 2018. A plant-centered diet was represented by higher A Priori Diet Quality Score (APDQS). A blood cholesterol-lowering diet was represented by lower Keys Score. Proportional hazards regression was used to calculate hazard ratios (HR). Higher APDQS showed a nutrient-dense composition that is low in saturated fat but high in fiber, vitamins and minerals. Keys Score and APDQS changes were each inversely associated with concurrent plasma low-density lipoprotein cholesterol (LDL-C) change. Over follow-up, 116 CHD and 80 stroke events occurred. LDL-C predicted CHD, but not stroke. APDQS, but not Keys Score, predicted lower risk of CHD and of stroke. Adjusted HRs (95% CIs) for each 1-SD higher APDQS were 0.73 (0.55–0.96) for CHD and 0.70 (0.50–0.99) for stroke. Neither low dietary fat nor low dietary carbohydrate predicted these events. Our findings support the ongoing shift in diet messages for cardiovascular prevention.

## 1. Introduction

Over recent decades, there has been a shift in focus from nutrient-based messages to recommendations about food groups and diet patterns [1]. The low fat recommendation, particularly, has been one of the most influential dietary messages ever delivered [2]. Low fat promulgated in the 1980 Dietary Guidelines for Americans (DGA) was adopted, which was influenced by the diet-heart hypothesis (Keys) [2,3]. In that hypothesis, especially saturated fat, contribute to elevated blood cholesterol, which thereby increases coronary heart disease (CHD) risk. The simplification in this recommendation was the assertion that reducing total fat would reduce saturated fat and, in general, lead to a more healthful serum lipid profile [4]. Starting in about 2010, healthy eating patterns have been incorporated into the Dietary Guidelines for Americans (DGA) with an emphasis on nutritionally rich plant foods [1]. Nevertheless, DGA maintain low saturated fat advice [5,6]. Over these years, a contrasting message has been to eat a diet low in carbohydrates. While never endorsed in the DGA, this idea has been widely noted [7,8].

A dietary recommendation can be seen as a set of rules for consumers about which foods to eat. Ideal study of a recommendation to follow a certain rule would be a randomized trial, giving instructions for how to follow a particular rule. Such studies are rare, especially with long-term follow-up [9]. An observational study is a naturalistic realization of a diet rule, examining who meets the criteria specified in the rule. For example, the rule in the low-fat message would be to eat little fat. In this sense, the percent of energy from fat represents adherence to this recommendation. Similarly, a diet pattern score would represent personal adherence to the dietary pattern. Therefore, we use the term “adherence” in our observational data as indicating what diet our participants actually ate, without knowledge of participant dietary intentions, and acknowledging that a wide variety of actual diets could meet, for example, a low-fat criterion. Thus, observational studies can examine the whole diet in people who actually follow the diet rule.

Our group previously showed that the A Priori Diet Quality Score (APDQS) was associated with cardiovascular disease (CVD), CHD, hypertensive-related CVD, and other clinical outcomes [10,11,12]. Expanding on our previous findings, the present study aimed to better understand how well different rules for choosing what to eat predicted CVD outcomes. We focused primarily on a comparative assessment between APDQS and a rule with a strong theoretical basis, namely a blood cholesterol-lowering diet (as operationalized by Keys Score, which explicitly incorporates lower saturated fat) in relation to CVD risk. The specific research questions were as follows. First, is consuming a cholesterol-lowering diet or a nutritionally rich plant-centered diet (as operationalized by the APDQS) associated with lower LDL-C and non-high-density lipoprotein cholesterol (non-HDL-C)? Second, do plasma LDL-C and non-HDL-C predict incident CHD and stroke? Third, which diet better predicts incident CHD and stroke? Fourth, we evaluated in parallel low fat and low carbohydrate intakes.

## 2. Materials and Methods

### 2.1. Study Design and Subjects

The Coronary Artery Risk Development in Young Adults (CARDIA) cohort study was initiated to examine the development and progression of CVD risk [13]. CARDIA enrolled 5115 Black and White adults from four US cities, aged 18–30 years in 1985–1986 (exam year 0 [Y0]), and able to walk on a treadmill when recruited. Participants in each field center included a balanced proportion by age, sex, race, and education. Participants have been contacted biannually for 32 years and had nine clinical examinations. Institutional review boards at all study sites approved the study, and all participants gave informed consent. Exclusion criteria for the current analyses included missing data at Y0 for LDL-C; implausible energy intakes (<800 or >8000 kcal/d for men and <600 or >6000 kcal/d for women); having CVD, diabetes or hypertension or receiving treatment for those conditions at Y0; missing covariates. No one took lipid-lowering medications until Y5 in this sample. After exclusion, the final sample for CVD outcomes analyses included 4701 participants, and the plasma lipids analyses included 3495 for 7-year change, 2360 for 13-year change, and 2824 for 20-year change.

### 2.2. Diet Assessment

Diet data were collected using an interviewer-administered diet history at Y0, Y7, and Y20. The reliability and validity of the questionnaire were established previously [14]. Trained interviewers asked the participants about food consumption over the previous month among 100 food categories and recorded open-ended responses of specific types of foods and beverages mentioned, their frequency of consumption, their unit or serving sizes, and preparation methods. Total energy and nutrient intake were estimated based on the Nutrition Data System for Research (NDSR, University of Minnesota, Minneapolis, MN, USA) [15].

The NDSR summarized foods into 166 food groups (the same in each exam), which CARDIA then collapsed into 46 food groups for the purpose of creating APDQS, a hypothesis-driven index. APDQS has been validated with varying degrees of predictive ability for obesity, diabetes, kidney function decline, myocardial infarction (MI), and mortality [10,11,12,16,17]. We calculated the diet quality score of plant-centered diets using APDQS. The 46 food groups were classified into beneficial (n = 20), adverse (n = 13), and neutral (n = 13) groups, according to their presumed influence on CVD. Beneficially rated food groups were ranked into quintiles and given positive values (0 [lowest quintile] to 4 [highest quintile]), whereas adversely rated food groups were ranked into their own quintiles and given reverse scores (4 [lowest quintile] to 0 [highest quintile]). Neutrally rated food groups were assigned scores of zero. The total APDQS range was 0–132. In the ADPQS, alcohol was not initially designed to have a U-shaped effect on CVDs. However, the mean alcohol intake in the CARDIA population was very low, and accordingly the cut-off point for the highest quintile of alcoholic beverage intake used to calculate the APDQS was very low (beer 1.10, wine 0.38, liquor 0.45 drinks/day in the year of examination 0 [Y0]). Therefore, we argued that the APDQS tends to regard light to moderate alcohol consumption as beneficial. The mean (SD) for beer, wine, and liquor in the highest quintile was 2.7 (2.2), 1.0 (1.1), and 1.3 (1.6) drinks/day, respectively.

Keys Score was expressed as: 1.35×(2× % energy from saturated fat − % energy from polyunsaturated fat) + 1.5×√(mg dietary cholesterol/1000 kcal), with higher values related to higher plasma cholesterol [18].

### 2.3. Ascertainment of CVD Events

The first occurrences of CHD or stroke were identified via annual follow-ups and subsequent medical record reviews. CHD included MI, non-MI acute coronary syndrome, and atherosclerotic CHD. Deaths were identified from biannual contact with family members and linkage to the National Death Index. When appropriate, the death certificate, autopsy report, and hospital records were requested with next-of-kin consent. The underlying nonfatal diagnosis or cause of death was adjudicated by two physicians or by committee consensus.

### 2.4. Fasting Plasma Lipid Measurements

Venous blood was drawn after a 12-h fast and sent to a central laboratory. Total cholesterol and HDL-C concentrations were measured using enzymatic reactions [19]. HDL-C was measured after dextran sulfate-magnesium precipitation of other lipoproteins [20], and LDL-C was calculated using the Friedewald equation [21]. Non-HDL-C was calculated as total cholesterol minus HDL-C.

### 2.5. Assessment of Covariates

All covariates were collected at baseline (Y0) and updated at each of the eight follow-up examinations. Information on age, race, educational level, smoking status, medical history, medication use was collected via a self-reported standardized questionnaire and a review of medication bottles. Physical activity levels were assessed through a validated interviewer-administered physical activity history questionnaire which inquired about the frequency of 13 physical activities, with the intensity-weighted summation over all activities expressed as exercise units [22]. Pack-years of smoking were calculated by multiplying the number of packs of cigarettes smoked per day by the duration of smoking in years. Weights and heights directly measured by trained staff were used to calculate body mass index (BMI; kg/m^2^).

### 2.6. Statistical Analyses

We used linear regression models to investigate the association between changes in APDQS and Keys Score (per 1 SD increase in each) over 7 (Y7–Y0), 13 (Y20–Y7), and 20 (Y20–Y0) years, and the dependent variables of concurrent changes in LDL-C and non-HDL-C. Y0 was the baseline for the 7- and 20-year changes, and Y7 for the 13-year changes. Models were adjusted for baseline LDL-C or non-HDL-C, baseline age, sex, race (White or Black), total energy intake (baseline and change), maximal educational attainment, parental history of CVD (yes or no), smoking pack-years (baseline and change), physical activity levels (baseline and change), time-varying use of lipid-lowering medications (yes or no), and BMI (baseline and change). Analyses were repeated after excluding individuals who had ever used lipid-lowering medications during each of the change periods.

We used Cox proportional hazards regression to estimate hazard ratios (HRs) and 95% confidence intervals (CIs) for CHD and stroke. These were evaluated separately for time-varying APDQS, Keys Score, LDL-C, and non-HDL-C, coded both as quintiles and as continuous variables. Y0 exposure variables predicted events from Y0 to Y7, the average of Y0 and Y7 exposure variables predicted events from after Y7 to Y20, and the average of Y0, Y7, and Y20 exposure variables predicted events from after Y20 to Y32. Note that if both Y7 and Y20 diet was missing, Y0 data was the predictor over all follow-up. The proportional hazards assumption was tested by including interaction terms between the exposures and the log of follow-up time in each model, and hazards were found to be proportional. Person-years were calculated from the date of baseline examination to the date of first reported CVD outcome of interest, death from any cause, or 31 August 2018−whichever came first. The model was adjusted for baseline age, sex, race, total energy intake, maximal educational attainment, parental history of CVD, smoking pack-years, physical activity level, use of lipid-lowering medications, and BMI; time-varying covariates were education, total energy intake, smoking, physical activity, use of lipid-lowering medications, and BMI. In separate models, the diet scores were adjusted for each other and lipid variables. Parallel analyses were performed using % energy from total fat and carbohydrate intake to represent other popular diet messages. Statistical significance of multiplicative interactions between each diet and lipid-lowering medications, race, sex, and BMI in association with CVD outcomes were assessed using Wald test. Statistical analyses were performed using SAS version 9.4 (SAS Institute Inc., Cary, NC, USA), with two-tailed significance set to 0.05.

## 3. Results

### 3.1. Study Population

At Y0, the mean ± SD was 62.7 ± 13.0 for APDQS and 48.0 ± 10.5 for Keys Score. Participants with higher APDQS tended to be older, female, White race, more active, more highly educated, and less likely to smoke than participants with lower APDQS (Table 1). They had lower BMI, lower LDL-C and non-HDL-C, and higher HDL-C compared with those in the lowest quintile. Participants with higher Keys Score had characteristics similar to those with lower APDQS, except for no physical activity difference. While extreme quintiles of APDQS were associated with somewhat lower range of saturated fat than were extreme quintiles of Keys Score, APDQS had the larger range of micronutrients and salting behavior.

### 3.2. Characteristics of Diet Indices

Detailed analysis of servings/day of the 46 food groups illuminates how high and low APDQS and Keys Scores were actuated (Table 2). With about 45 servings/day eaten, those in the highest vs. lowest APDQS quintiles ate 21 vs. 9 servings/day of beneficially rated foods. In contrast, those in the lowest vs. highest Keys Score quintiles ate 16 vs. 13 servings/day of beneficially rated foods. The pattern for adversely rated foods also favored APDQS (24.92 in the lowest vs. 13.42 in the highest), while it was unfavored for Keys score (14.49 in the lowest vs. 23.19 in the highest). Despite the fact that the Keys score was driven by dietary lipids and cholesterol, the data showed that high Keys score diet was generally characterized by a diet low in nutritionally rich plant foods but high in unhealthy plant foods and red and processed meats. Overall, the data indicate that high APDQS was largely driven by nutritionally rich plant foods, and nutritionally rich plant foods make a large majority of daily servings of those who had a high APDQS.

The correlation between Y0 APDQS and Y0 Keys Score was −0.31 (*p*-value < 0.001).

### 3.3. Association of Diet Score with Plasma Lipids

At Y0, the mean ± SD was 2.82 ± 0.80 mmol/L for LDL-C and 3.19 ± 0.87 mmol/L for non-HDL-C. Every 1 SD (13 points) increase in APDQS was associated with concurrent LDL-C reduction (−0.05 ± 0.02, *p* = 0.007) and non-HDL-C reduction (−0.06 ± 0.02, *p* < 0.001) over 20 years, and similarly for 7- and 13-year changes (Table 3).

Each 1 SD (11 points) decrease in Keys Score over 20 years was associated with reduced LDL-C (−0.05 ± 0.01, *p* < 0.001) and reduced non-HDL-C (−0.06 ± 0.01, *p* < 0.001), and similarly for 7- and 13-year changes. These associations were slightly attenuated after excluding any lipid-lowering medication users during the period evaluated (data not shown).

### 3.4. Association of Diet Score and Plasma Lipids with CVD Outcomes

During the median 32-year follow-up, 116 incident CHD, and 80 stroke events occurred. For CHD, the HRs (95% CIs) for every 1 SD higher were as follows: 0.73 (0.55–0.96) for APDQS, 0.86 (0.68–1.08) for Keys Score, 1.70 (1.42–2.03) for LDL-C, and 1.74 (1.46–2.08) for non-HDL-C (Table 4). The association between APDQS and CHD persisted after adjustment for LDL-C, non-HDL-C or Keys Score (Supplementary Materials, Table S1). APDQS was associated with risk of stroke 0.70 (0.50–0.99; per 1 SD), but Keys Score, LDL-C, and non-HDL-C were not. Adjustment for LDL-C, non-HDL-C, or Keys Score did not substantially alter this finding. The associations of APDQS (or Keys Score) with CHD and stroke risk did not vary by lipid-lowering medication use, race, or BMI (P-interaction was >0.05 for each).

In analyses of other simple messages that are currently popular, neither total fat nor total carbohydrate intake were associated with risk of CHD or stroke (Table 5), although total fat was minimally correlated with LDL-C (Supplementary Materials, Table S2).

## 4. Discussion

Expanding on our previous finding that APDQS predicted incident CVD outcomes [10], we conducted a head-to-head comparison of predictiveness for CHD and stroke of APDQS vs. Keys score (strongly based in theory and representing a blood cholesterol-lowering diet), total fat restriction, and total carbohydrate restriction. In so doing, we interpreted observed long-term diet features as indicative of different dietary recommendations. There are three main findings of this 32-year prospective study starting with a younger, generally healthy, community-based sample. First, LDL-C predicted incident CHD, consistent with historical assertions within the diet-heart hypothesis that is reflected in the Keys Score [3]. Second, a decrease in Keys Score, primarily driven by low saturated fat intake, was associated with concurrent reductions in both LDL-C and non-HDL-C, again in line with historical assertions. Similarly, APDQS, discouraging high saturated fat foods to some extent, but more importantly emphasizing nutritionally rich plant foods, was inversely associated with concurrent changes in LDL-C and non-HDL-C. Third, we clarified that the diet based on Keys Score has little association with risk of incident CHD and stroke. In contrast, a nutritionally rich plant-centered diet represented by high APDQS was associated with a 27% and 30% lower risk of incident CHD and stroke, respectively, per 1 SD higher of APDQS. Notably, only APDQS had an inverse association with incident stroke. Although restricting total fat and restricting total carbohydrate are dietary messages that persist in the general population, neither was related to incident CHD or stroke.

Publicly promoted messages have been oversimplified, such as “low in saturated fat”, “low in total fat”, and “low in carbohydrates”. They do not clearly specify whether to eat foods that are low in the nutrient that is the focus of the message but may contain other constituents that should be encouraged or discouraged. Particularly, saturated fat intake has long been considered to be atherogenic, and that its reduction may decrease the risk of CHD [3]. The 2020–2025 DGA recommends that saturated fats should comprise <10% energy, and the AHA/ACC recommends 5–6% [6,23]. Yet, results from observational studies and randomized controlled trials (RCTs) are contradictory and inconclusive regarding such an association between saturated fat intake and the risk of CVD [24,25]. In addition, meta-analysis of prospective studies showed that PUFA or MUFA intake was not associated with risk of CVD, although an inverse association between PUFA and CVD risk was observed in a subgroup analysis of studies followed up for more than 10 years [25]. Results from RCTs and prospective studies generally suggested that replacing saturated fats with PUFAs, MUFAs derived from plant foods (but not from animal sources), or whole grains may reduce the risk of CHD [24,26,27,28,29]. However, it is in practice challenging to separate specific types of fats from other constituents in food because saturated fat is always part of food.

Our data support the a priori hypothesis that LDL-C is causally related to CHD, but the LDL-C association with stroke is not as strong as with CHD [30,31]. We found that higher APDQS and lower Keys Scores were similarly associated with decreases in LDL-C and non-HDL-C. However, only high APDQS predicted CHD and stroke and this prediction may be partly through LDL-C lowering. As actually observed, diet patterns based on Keys score tended to lack numerous acaloric antioxidant nutrients and bioactive phytochemicals, which could explain its lack of association with CHD or stroke. APDQS codifies many of the guidelines in DGA, with some additional classifications and slightly different principles. In APDQS, no one food choice is very influential, as there are many other alternative and eating opportunities. Our assertion is that plant-centered diet pattern recommendations can achieve diets low in saturated fat (e.g., through recommending plant foods and lean and low-fat animal foods), added sugars, and other components that are in accordance with the current dietary guidelines without explicit mention of the nutrient. Furthermore, APDQS itself takes into account the food substitution effect to some extent. For instance, APDQS emphasizes lean and low-fat animal foods (vs. high-fat animal foods), low fat dairy (vs. whole-fat dairy), and whole grains (vs. highly refined sweetened “foods/beverages). Overall, our findings emphasize the importance of considering more than just a single or a combination of nutrients for the prevention of CVD. This thinking is closely allied with the “3V” rule of Fardet and Rock, emphasizing Végétal (plant), Vrai (real) and Varié (varied, if possible organic, local and seasonal) aspects of food choice [32].

Various mechanisms may be involved in providing the cardiovascular benefits of a plant-centered diet. By eating a variety of plant-based foods, including fruits, vegetables, whole grains, legumes, and nuts, individuals can consume an adequate set of vitamins, minerals, fibers, and phytochemicals, all of which interact with each other to reduce the risk of CVD through a number of their properties—they are anti-oxidative; anti-inflammatory; anti-hypertensive; anti-thrombotic; improving glucose control and cholesterol concentrations; as well as their functional properties of low glycemic load and energy density [33]. In contrast, animal-based foods, especially red meats may be harmful partly due to the following characteristics that have been associated with an increased risk of CVD: increased LDL-C and apolipoprotein B, independently of saturated fat [34]; high in dietary haem iron—which increases oxidative stress level [35]; and is high in dietary precursors of trimethylamine-N-oxide [36].

The strengths of this study include its prospective study design following healthy, young adults over a 32-year period, its high retention rate among survivors, and its detailed and long-term pattern measurements of overall diet quality and covariates. The present study, however, has limitations. Given its observational nature, residual and unmeasured confounding biases are possible, although dynamic changes in important risk factors during the follow-ups were controlled for in the time-varying analyses. The generalizability of findings to other populations may be limited.

## 5. Conclusions

Our head-to-head comparison of different diet criteria supports the ongoing shift in diet messaging from nutrients to food-based dietary patterns. It finds that several simple nutrient-based rules for choosing what to eat are not associated with incident CHD, even when the message (i.e., low Keys score) has a strong theoretical basis. Such messages are incomplete in that they do not provide guidance about eating or not eating a wide variety of foods. In this sense, the current public health message to reduce intake of saturated fat to decrease the risk of CVD may not be helpful. Our findings provide formal support for promoting total diet quality, with nutritionally rich plant foods at its center.

## Figures and Tables

**Table 1:** Baseline characteristics (Y0) of the participants according to quintiles of the Y0 APDQS and Keys Score ^a.^

	**APDQS**					
**Baseline Characteristics**	**Quintile 1** **(≤51)**	**Quintile 2** **(52–58)**	**Quintile 3** **(59–65)**	**Quintile 4** **(66–74)**	**Quintile 5** **(≥75)**	***p*-Value ^b^**
**Hypothesized Direction of** **the Score**	**Lower** **Plant-Centered Diet Quality**				**Higher** **Plant-Centered Diet Quality**	
Participants, n	975	945	920	954	907	
Age, mean (SD), y	23 ± 3.69	24.3 ± 3.71	25 ± 3.6	25.6 ± 3.26	26.3 ± 2.95	<0.001
Female, No (%)	485 (49.7)	499 (52.8)	491 (53.4)	515 (54)	604 (66.6)	<0.001
Black race, No (%)	764 (78.4)	634 (67.1)	479 (52.1)	351 (36.8)	123 (13.6)	<0.001
Maximal educational attainment, mean (SD), grades ^c^	14.1 ± 2.34	14.6 ± 2.48	15.2 ± 2.58	16 ± 2.58	17.0 ± 2.32	<0.001
Physical activity, mean (SD), EU ^d^	359 ± 273	373 ± 296	407 ± 288	424 ± 275	521 ± 311	<0.001
Current smoker, No (%)	314 (32.5)	325 (34.6)	298 (32.5)	261 (27.6)	184 (20.4)	<0.001
Parental history of CVD, No (%)	370 (38)	367 (38.8)	390 (42.4)	343 (36)	365 (40.2)	0.06
BMI, mean (SD), kg/m^2^	24.4 ± 5.34	24.8 ± 5.24	24.9 ± 5.10	24.3 ± 4.6	23.4 ± 3.68	<0.001
Total cholesterol, mean (SD), mmol/L	4.51 ± 0.88	4.58 ± 0.86	4.56 ± 0.87	4.61 ± 0.84	4.56 ± 0.81	0.040
LDL-C, mean (SD), mmol/L	2.80 ± 0.82	2.85 ± 0.80	2.85 ± 0.84	2.87 ± 0.78	2.75 ± 0.75	0.005
HDL-C, mean (SD), mmol/L	1.34 ± 0.33	1.37 ± 0.34	1.35 ± 0.33	1.38 ± 0.35	1.46 ± 0.34	<0.001
Non-HDL-C, mean (SD), mmol/L	3.16 ± 0.88	3.21 ± 0.87	3.21 ± 0.91	3.24 ± 0.85	3.08 ± 0.82	<0.001
Alcohol intake, mean (SD), drinks/d	0.44 ± 1.07	0.77 ± 1.47	0.89 ± 1.72	1.00 ± 1.39	0.95 ± 1.09	<0.001
Energy intake, mean (SD), kcal/d	3176 ± 1386	2883 ± 1403	2881 ± 1466	2687 ± 1238	2449 ± 1035	<0.001
Total fat, mean (SD), % of energy	38.6 ± 5.68	38.2 ± 5.82	38.5 ± 5.73	37.4 ± 5.79	35.5 ± 6.40	<0.001
Total carbohydrate, mean (SD), % of energy	46.9 ± 7.23	45.8 ± 7.51	45.0 ± 7.08	45.3 ± 7.27	47.2 ± 7.76	<0.001
Total protein, mean (SD), % of energy	13.7 ± 2.36	14.5 ± 2.46	14.9 ± 2.46	15.3 ± 2.70	15.8 ± 2.85	<0.001
Saturated fat, mean (SD), % of energy	15.0 ± 2.81	14.5 ± 2.81	14.5 ± 2.87	13.9 ± 2.90	12.8 ± 3.06	<0.001
MUFA, mean (SD), % of energy	14.7 ± 2.42	14.4 ± 2.45	14.3 ± 2.43	13.6 ± 2.33	12.5 ± 2.75	<0.001
PUFA, mean (SD), % of energy	6.24 ± 1.69	6.63 ± 1.90	7.02 ± 1.99	7.18 ± 2.32	7.40 ± 2.47	<0.001
Dietary cholesterol, mean (SD), mg/1000 kcal	178 ± 66.9	177 ± 72.9	170 ± 64.3	162 ± 58.0	147 ± 59.0	<0.001
Fiber, mean (SD), g/1000 kcal	1.37 ± 0.49	1.66 ± 0.60	1.95 ± 0.68	2.35 ± 0.85	3.21 ± 1.35	<0.001
Vitamin A, mean (SD), IU/1000 kcal	2155 ± 1601	2582 ± 1536	3175 ± 3612	4014 ± 3178	6717 ± 6619	<0.001
Vitamin C, mean (SD), mg/1000 kcal	50.2 ± 30.6	56.4 ± 34.8	64.9 ± 39.2	74.6 ± 42.5	94.1 ± 51.1	<0.001
Folate, mean (SD), µg/1000 kcal	95.3 ± 43.8	142 ± 735	126 ± 104	157 ± 251	233 ± 421	<0.001
Calcium, mean (SD), mg/1000 kcal	390 ± 150	413 ± 162	439 ± 182	464 ± 176	513 ± 163	<0.001
Potassium, mean (SD), mg/1000 kcal	1061 ± 217	1188 ± 266	1312 ± 282	1456 ± 306	1749 ± 439	<0.001
Magnesium, mean (SD), mg/1000 kcal	105 ± 22.0	119 ± 27.7	133 ± 32.6	149 ± 33.6	173 ± 41.0	<0.001
Sodium, mean (SD), mg/1000 kcal ^e^	1441 ± 261	1466 ± 277	1508 ± 305	1528 ± 306	1523 ± 329	<0.001
Almost always use salt in cooking at home, No (%)	442 (45.3)	389 (41.2)	309 (33.6)	245 (25.7)	140 (15.4)	<0.001
Almost always use salt at the table, No (%)	212 (21.7)	149 (15.8)	140 (15.2)	101 (10.6)	60 (6.6)	<0.001
	**Keys Score**					
**Baseline characteristics**	**Quintile 1** **(≤ 39)**	**Quintile 2** **(40–45)**	**Quintile 3** **(46–50)**	**Quintile 4** **(51–56)**	**Quintile 5** **(≥57)**	***p*-Value ^b^**
**Hypothesized** **direction of the score**	**Blood cholesterol-lowering diet**				**Blood cholesterol-increasing diet**	
Participants, n	940	940	941	940	940	
Age, mean (SD), y	25.3 ± 3.56	25 ± 3.54	24.8 ± 3.64	24.6 ± 3.68	24.3 ± 3.74	<0.001
Female, No (%)	615 (65.4)	546 (58.1)	497 (52.8)	485 (51.6)	451 (48)	<0.001
Black race, No (%)	426 (45.3)	446 (47.5)	471 (50.1)	493 (52.5)	515 (54.8)	<0.001
Maximal educational attainment, mean (SD), grades ^c^	15.7 ± 2.60	15.6 ± 2.6	15.4 ± 2.69	15.2 ± 2.71	14.9 ± 2.60	<0.001
Physical activity, mean (SD), EU ^d^	420 ± 293	414 ± 293	417 ± 302	417 ± 295	411 ± 286	0.97
Current smoker, No (%)	213 (22.8)	275 (29.4)	281 (30.1)	281 (30)	332 (35.8)	<0.001
Parental history of CVD, No (%)	366 (38.9)	380 (40.4)	361 (38.4)	357 (38)	371 (39.5)	0.83
BMI, mean (SD), kg/m^2^	24.7 ± 5.20	24.7 ± 5.07	24.2 ± 4.66	24.2 ± 4.63	24.0 ± 4.72	0.007
Total cholesterol, mean (SD), mmol/L	4.56 ± 0.86	4.53 ± 0.84	4.56 ± 0.84	4.64 ± 0.88	4.56 ± 0.84	0.11
LDL-C, mean (SD), mmol/L	2.80 ± 0.80	2.80 ± 0.80	2.80 ± 0.79	2.87 ± 0.82	2.82 ± 0.80	0.13
HDL-C, mean (SD), mmol/L	1.37 ± 0.33	1.37 ± 0.33	1.40 ± 0.35	1.38 ± 0.35	1.37 ± 0.35	0.18
Non-HDL-C, mean (SD), mmol/L	3.17 ± 0.87	3.16 ± 0.87	3.16 ± 0.86	3.24 ± 0.89	3.19 ± 0.85	0.20
Alcohol intake, mean (SD), drinks/d	0.94 ± 1.93	0.86 ± 1.36	0.83 ± 1.34	0.76 ± 1.12	0.64 ± 0.96	<0.001
Energy intake, mean (SD), kcal/d	2439 ± 1135	2746 ± 1286	2926 ± 1370	2934 ± 1354	3054 ± 1440	<0.001
Total fat, mean (SD), % of energy	32.5 ± 6.75	36.2 ± 5.04	38.0 ± 4.40	39.5 ± 4.03	42.3 ± 4.37	<0.001
Total carbohydrate, mean (SD), % of energy	52.0 ± 8.50	47.8 ± 6.41	45.6 ± 5.68	43.9 ± 5.48	40.9 ± 5.46	<0.001
Total protein, mean (SD), % of energy	14.0 ± 3.17	14.4 ± 2.47	14.8 ± 2.27	15.1 ± 2.33	15.6 ± 2.66	<0.001
Saturated fat, mean (SD), % of energy	10.5 ± 1.96	12.8 ± 1.26	14.1 ± 1.15	15.5 ± 1.19	18.0 ± 2.10	<0.001
MUFA, mean (SD), % of energy	11.9 ± 2.79	13.5 ± 2.26	14.2 ± 2.15	14.7 ± 2.12	15.3 ± 2.16	<0.001
PUFA, mean (SD), % of energy	7.70 ± 2.96	7.21 ± 2.10	6.90 ± 1.80	6.50 ± 1.59	6.10 ± 1.44	<0.001
Dietary cholesterol, mean (SD), mg/1000 kcal	113 ± 36.3	144 ± 38.9	165 ± 43.3	184 ± 51.3	228 ± 81.2	<0.001
Fiber, mean (SD), g/1000 kcal	2.77 ± 1.48	2.19 ± 0.91	2.02 ± 0.86	1.84 ± 0.73	1.67 ± 0.76	<0.001
Vitamin A, mean (SD), IU/1000 kcal	5072 ± 6808	3677 ± 3419	3494 ± 3277	3141 ± 2437	3106 ± 2429	<0.001
Vitamin C, mean (SD), mg/1000 kcal	90.4 ± 59.2	72.0 ± 40.2	64.1 ± 35.3	59.6 ± 31.0	52.7 ± 32.4	<0.001
Folate, mean (SD), µg/1000 kcal	179 ± 270	156 ± 641	148 ± 305	126 ± 170	140 ± 441	0.06
Calcium, mean (SD), mg/1000 kcal	403 ± 178	411 ± 151	434 ± 159	454 ± 159	513 ± 189	<0.001
Potassium, mean (SD), mg/1000 kcal	1518 ± 524	1346 ± 353	1322 ± 351	1287 ± 308	1269 ± 317	<0.001
Magnesium, mean (SD), mg/1000 kcal	149 ± 50.6	136 ± 37.0	133 ± 37.3	130 ± 34.0	129 ± 34.1	<0.001
Sodium, mean (SD), mg/1000 kcal ^e^	1449 ± 362	1477 ± 286	1498 ± 277	1509 ± 270	1533 ± 279	<0.001
Almost always use salt in cooking at home, No (%)	266 (28.3)	301 (32)	287 (30.5)	326 (34.7)	345 (36.7)	<0.001
Almost always use salt at the table, No (%)	109 (11.6)	117 (12.5)	140 (14.9)	138 (14.7)	158 (16.8)	<0.001

APDQS, A Priori Diet Quality Score; BMI, body mass index; CVD, cardiovascular disease; LDL-C, low-density lipoprotein cholesterol; MUFA, monounsaturated fatty acids; non-HDL-C, non-high-density lipoprotein cholesterol; PUFA, polyunsaturated fatty acids. SI conversion factors: To convert cholesterol to millimoles per liter, multiply by 0.0259. ^a^ Total score sums the 46 components (possible scores 0–132, with a range of 35–95 in these data), with higher scores representing a nutritionally rich, plant-centered diet. A one-point increment represents a one-category shift in the presumed favorable direction. Beneficially rated food group includes fruit, avocado, beans/legumes, green vegetables, yellow vegetables, tomatoes, other vegetables, nuts and seeds, soy products, whole grains, vegetable oil, fatty fish, lean fish, poultry, alcohol (beer, wine, and liquor), coffee, tea, and low-fat milk/cheese/yogurt. In practice, the amount of alcohol consumed was rarely more than a moderate level. Adversely rated food group includes fried potatoes, grain dessert, salty snacks, pastries, sweets, high-fat red meats, processed meats, organ meats, fried fish/poultry, sauces, soft drink, whole-fat milk/cheese/yogurt, and butter. Neutrally rated food group includes potatoes, refined grains, margarine, chocolate, meal replacements, pickled foods, sugar substitutes, lean meats, shellfish, eggs, soups, diet drinks, and fruit juices. ^b^ Evaluated with chi-square tests for categorical variables and ANOVA for continuous variables. ^c^ Cumulative data through Y30. ^d^ Exercise units, physical activity score derived from the CARDIA physical activity history. ^e^ Represents only for the amount of sodium in food before cooking or table salt.

**Table 2:** Mean intake (serving/day) of 46 individual food groups according to quintiles of the APDQS and Keys Score at Y0.

	APDQS			Keys Score			Difference of Differences ^a^
Food Group (Mean ± SD)	Quintile 1	Quintile 5	Difference (Quintile 5-Quintile 1)	Quintile 1	Quintile 5	Difference (Quintile 1-Quintile 5)	
Beneficially rated food groups							
1. Fruit	0.94 ± 1.36	2.06 ± 1.66	1.12	1.74 ± 1.83	1.12 ± 1.27	0.62	0.50
2. Avocado	0.01 ± 0.10	0.24 ± 0.47	0.23	0.10 ± 0.37	0.06 ± 0.21	0.04	0.19
3. Beans and legumes	0.17 ± 0.35	0.24 ± 0.40	0.07	0.22 ± 0.44	0.17 ± 0.34	0.05	0.02
4. Green vegetables	0.13 ± 0.25	0.91 ± 1.27	0.78	0.57 ± 1.07	0.28 ± 0.43	0.29	0.49
5. Yellow vegetables	0.08 ± 0.21	0.61 ± 0.98	0.53	0.41 ± 1.04	0.18 ± 0.40	0.23	0.30
6. Tomato	0.32 ± 0.30	0.73 ± 0.65	0.41	0.52 ± 0.64	0.43 ± 0.41	0.09	0.32
7. Other vegetables	1.43 ± 1.18	2.88 ± 2.17	1.45	2.35 ± 2.13	1.77 ± 1.48	0.58	0.87
8. Nuts and seeds	0.40 ± 0.85	1.13 ± 1.66	0.73	1.03 ± 1.98	0.56 ± 0.89	0.47	0.26
9. Soy products	0.09 ± 0.44	0.47 ± 1.07	0.38	0.25 ± 0.68	0.22 ± 0.78	0.03	0.35
10. Whole grains	1.02 ± 1.32	2.14 ± 1.75	1.12	1.71 ± 1.74	1.40 ± 1.50	0.31	0.81
11. Vegetable oil	0.89 ± 1.26	2.04 ± 2.09	1.15	1.52 ± 1.86	1.43 ± 1.81	0.09	1.06
12. Fatty fish	0.01 ± 0.07	0.08 ± 0.25	0.07	0.03 ± 0.14	0.03 ± 0.17	0	0.07
13. Lean fish	0.44 ± 0.92	1.02 ± 1.50	0.58	0.85 ± 1.48	0.50 ± 0.80	0.35	0.23
14. Poultry	1.06 ± 1.15	1.41 ± 1.71	0.35	1.24 ± 1.74	1.11 ± 1.33	0.13	0.22
15. Beer	0.30 ± 0.88	0.47 ± 0.72	0.17	0.50 ± 1.32	0.37 ± 0.71	0.13	0.04
16. Wine	0.04 ± 0.23	0.30 ± 0.48	0.26	0.19 ± 0.72	0.13 ± 0.33	0.06	0.20
17. Liquor	0.10 ± 0.51	0.18 ± 0.46	0.08	0.25 ± 0.83	0.15 ± 0.35	0.10	−0.02
18. Coffee	0.45 ± 1.62	1.84 ± 2.71	1.39	1.00 ± 2.34	1.34 ± 2.65	−0.34	1.73
19. Tea	0.33 ± 1.3	0.85 ± 2.19	0.52	0.74 ± 2.68	0.38 ± 1.46	0.36	0.16
20. Low-fat milk/Cheese/Yogurt	0.57 ± 1.10	1.66 ± 1.53	1.09	1.08 ± 1.39	1.17 ± 2.06	−0.09	1.18
Subtotal	8.78	21.26	12.48	16.3	12.8	3.50	8.98
Neutrally rated food groups							
1. Potatoes	0.38 ± 0.53	0.34 ± 0.39	−0.04	0.35 ± 0.53	0.40 ± 0.68	−0.05	0.01
2. Refined grains	5.64 ± 3.27	3.11 ± 2.17	−2.53	3.58 ± 2.69	4.44 ± 2.89	−0.86	−1.67
3. Margarine	1.66 ± 2.41	1.36 ± 2.06	−0.30	1.86 ± 2.54	1.13 ± 2.24	0.73	−1.03
4. Chocolate	0.22 ± 0.38	0.12 ± 0.22	−0.10	0.14 ± 0.29	0.21 ± 0.45	−0.07	−0.03
5. Meal replacements	0.01 ± 0.13	0.01 ± 0.07	0	0.01 ± 0.10	0.01 ± 0.05	0	0
6. Pickled foods	0.29 ± 0.73	0.40 ± 0.63	0.11	0.32 ± 0.54	0.33 ± 0.80	−0.01	0.12
7. Sugar substitutes	0.01 ± 0.10	0.13 ± 0.54	0.12	0.09 ± 0.46	0.05 ± 0.44	0.04	0.08
8. Lean red meats	0.88 ± 1.88	0.48 ± 0.76	−0.40	0.52 ± 0.85	0.92 ± 1.96	−0.40	0
9. Shellfish	0.13 ± 0.38	0.27 ± 0.42	0.14	0.20 ± 0.49	0.20 ± 0.39	0	0.14
10. Eggs	0.79 ± 0.80	0.49 ± 0.52	−0.30	0.29 ± 0.33	1.08 ± 0.98	−0.79	0.49
11. Soups	0.02 ± 0.06	0.04 ± 0.07	0.02	0.04 ± 0.09	0.04 ± 0.11	0	0.02
12. Diet soft drinks	0.11 ± 0.60	0.68 ± 1.44	0.57	0.52 ± 1.52	0.29 ± 0.90	0.23	0.34
13. Fruit juice	1.81 ± 2.35	1.87 ± 2.30	0.06	2.30 ± 3.21	1.46 ± 1.64	0.84	−0.78
Subtotal	11.95	9.30	−2.65	10.22	10.56	−0.34	−2.31
Adversely rated food groups							
1. Fried potatoes	0.53 ± 0.56	0.15 ± 0.24	−0.38	0.25 ± 0.44	0.35 ± 0.41	−0.10	−0.28
2. Grain desserts	0.98 ± 1.30	0.46 ± 0.66	−0.52	0.52 ± 0.69	0.67 ± 1.09	−0.15	−0.37
3. Salty snacks	0.03 ± 0.12	0.04 ± 0.18	0.01	0.06 ± 0.31	0.03 ± 0.10	0.03	−0.02
4. Pastries	1.24 ± 1.16	0.64 ± 0.72	−0.60	0.66 ± 0.95	1.10 ± 1.18	−0.44	−0.16
5. Sweets	1.98 ± 2.36	0.95 ± 1.36	−1.03	1.32 ± 1.94	1.89 ± 2.50	−0.57	−0.46
6. High-fat red meats	2.86 ± 2.11	1.13 ± 1.57	−1.73	1.48 ± 2.15	2.54 ± 2.14	−1.06	−0.67
7. Processed meats	1.21 ± 1.25	0.34 ± 0.66	−0.87	0.52 ± 0.80	1.02 ± 1.22	−0.50	−0.37
8. Organ meats	0.06 ± 0.19	0.02 ± 0.07	−0.04	0.03 ± 0.13	0.06 ± 0.17	−0.03	−0.01
9. Fried poultry and fish	0.14 ± 0.84	0.06 ± 0.49	−0.08	0.09 ± 0.66	0.12 ± 0.80	−0.03	−0.05
10. Sauces	4.61 ± 4.13	4.70 ± 9.39	0.09	4.57 ± 9.08	4.30 ± 5.69	0.27	−0.18
11. Soft drinks	2.69 ± 2.60	0.42 ± 0.72	−2.27	1.70 ± 2.74	1.14 ± 1.35	0.56	−2.83
12. Whole-fat milk/Cheese/Yogurt	2.53 ± 2.37	1.36 ± 1.34	−1.17	0.89 ± 0.97	3.29 ± 3.31	−2.40	1.23
13. Butter	6.06 ± 4.72	3.15 ± 3.14	−2.91	2.40 ± 2.35	6.68 ± 5.60	−4.28	1.37
Subtotal	24.92	13.42	−11.5	14.49	23.19	−8.70	−2.80
Grand total	45.65	43.98	−1.67	41.01	46.55	−5.54	3.87

^a^ Positive value favors APDQS for beneficial, negative value favors APDQS for adverse.

**Table 3:** Association between change in the APDQS and Keys Score and concurrent changes in LDL-C and Non-HDL-C ^a.^

	Adjusted Mean Change for Each 1 SD Increment ^b^					
	APDQS Change			Keys Score Change		
	*ß*	SE	*p*-Value	*ß*	SE	*p*-Value
LDL-C, mmol/L						
7-year change (mean age from 25 y to 32 y), n = 3495	−0.02	0.01	0.09	−0.02	0.01	0.023
13-year change (mean age from 32 y to 45 y), n = 2360	−0.07	0.02	<0.001	−0.07	0.01	<0.001
20-year change (mean age from 25 y to 45 y), n = 2824	−0.05	0.02	0.007	−0.05	0.01	<0.001
Non-HDL-C, mmol/L						
7-year change (mean age from 25 y to 32 y), n = 3495	−0.03	0.01	0.019	−0.03	0.01	0.023
13-year change (mean age from 32 y to 45 y), n = 2360	−0.09	0.02	<0.001	−0.08	0.01	<0.001
20-year change (mean age from 25 y to 45 y), n = 2824	−0.06	0.02	<0.001	−0.06	0.01	<0.001

APDQS, A Priori Diet Quality Score; LDL-C, low-density lipoprotein cholesterol; non-HDL-C, non-high-density lipoprotein cholesterol. ^a^ Each row is a separate linear regression. Model was adjusted for baseline LDL-C (or non-HDL-C), baseline age, sex, race (White or Black), total energy intake (baseline and change), maximal educational attainment, parental history of CVD (yes or no), pack-years of smoking (baseline and change), physical activity level (baseline and change), use of lipid-lowering medications (yes or no), and BMI (baseline and change).^b^ 1 SD changes were 13 for APDQS, and −11 for Keys Score.

**Table 4:** Multivariable-adjusted HRs (95% CIs) ^a^ of incident CHD and stroke according to quintiles of the time-varying average APDQS, Keys Score, LDL-C, and non-HDL-C ^b^.

	Quintile 1	Quintile 2	Quintile 3	Quintile 4	Quintile 5	Per 1 SD Change ^c^	P for Trend ^d^
CHD (Y0-Y32)							
APDQS							
Range	≤54.6	54.7–61.5	61.6–68	68.1–75.7	≥75.8		
Unadjusted cumulative incidence % (n/N)	3.6 (34/934)	3.2 (30/950)	2.4 (23/961)	2.2 (20/902)	0.9 (9/954)		
Adjusted HR	1.00 (ref)	0.67 (0.4–1.12)	0.66 (0.38–1.15)	0.63 (0.34–1.17)	0.38 (0.17–0.86)	0.73 (0.55–0.96)	0.023
Keys Score							
Range	≤36.2	36.3–40.9	41.0–45.0	45.1–50.5	≥50.6		
Unadjusted cumulative incidence % (n/N)	1.7 (16/940)	1.1 (10/940)	3.1 (29/941)	2.7 (25/940)	3.8 (36/940)		
Adjusted HR	0.66 (0.35–1.22)	0.54 (0.29–1.02)	0.95 (0.57–1.6)	0.81 (0.48–1.38)	1.00 (ref)	0.86 (0.68–1.08)	0.20
LDL-C							
Range (mmol/L)	≤2.22	2.23–2.59	2.60–2.96	2.97–3.39	≥3.40		
Unadjusted cumulative incidence % (n/N)	0.5 (5/940)	1.4 (13/942)	2.1 (20/939)	3.2 (30/940)	5.1 (48/940)		
Adjusted HR	1.00 (ref)	2.45 (0.87–6.91)	3.65 (1.37–9.78)	5.06 (1.95–13.13)	6.42 (2.52–16.37)	1.70 (1.42–2.03)	<0.001
Non-HDL-C							
Range (mmol/L)	≤2.58	2.59–3.00	3.01–3.40	3.41–3.91	≥3.92		
Unadjusted cumulative incidence % (n/N)	0.4 (4/940)	1.1 (10/940)	1.9 (18/941)	3.2 (30/940)	5.7 (54/940)		
Adjusted HR	1.00 (ref)	2.43 (0.76–7.75)	3.66 (1.22–10.98)	6.40 (2.24–18.3)	8.59 (3.05–24.24)	1.74 (1.46–2.08)	<0.001
Stroke (Y0-Y32)							
APDQS							
Range	≤54.6	54.7–61.5	61.6–68	68.1–75.7	≥75.8		
Unadjusted cumulative incidence % (n/N)	3.5 (33/935)	1.7 (16/948)	1.4 (13/961)	1.2 (11/902)	0.7 (7/955)		
Adjusted HR	1.00 (ref)	0.44 (0.24–0.82)	0.43 (0.21–0.85)	0.61 (0.29–1.27)	0.55 (0.21–1.45)	0.70 (0.50–0.99)	0.043
Keys Score							
Range	≤36.2	36.3-40.8	40.9-44.9	45.0-50.5	≥50.6		
Unadjusted cumulative incidence % (n/N)	1.3 (12/940)	1.8 (17/940)	1.3 (12/941)	2.1 (20/940)	2.0 (19/940)		
Adjusted HR	0.81 (0.38–1.73)	1.21 (0.63–2.34)	0.59 (0.27–1.29)	1.09 (0.57–2.09)	1.00 (ref)	0.93 (0.70–1.23)	0.59
LDL-C							
Range (mmol/L)	≤2.22	2.23–2.59	2.60–2.96	2.97–3.39	≥3.40		
Unadjusted cumulative incidence % (n/N)	1.5 (14/940)	1.7 (16/941)	1.4 (13/940)	1.9 (18/940)	2.0 (19/940)		
Adjusted HR	1.00 (ref)	1.41 (0.67–2.96)	1.10 (0.50–2.44)	1.38 (0.65–2.90)	1.22 (0.58–2.60)	1.14 (0.90–1.45)	0.27
Non-HDL-C							
Range (mmol/L)	≤2.58	2.59–3.00	3.01–3.40	3.41–3.91	≥3.92		
Unadjusted cumulative incidence % (n/N)	1.4 (13/940)	1.5 (14/940)	1.5 (14/941)	1.9 (18/940)	2.2 (21/940)		
Adjusted HR	1.00 (ref)	1.44 (0.66–3.14)	1.25 (0.56–2.77)	1.52 (0.71–3.28)	1.54 (0.71–3.34)	1.23 (0.97–1.56)	0.09

APDQS, A Priori Diet Quality Score; CHD, coronary heart disease; CI, confidence interval; HR, hazard ratio; LDL-C, low-density lipoprotein cholesterol; non-HDL, non-high-density lipoprotein cholesterol; SD, standard deviation. ^a^ Each row is a separate proportional hazards regression. Model was adjusted for Y0 age, sex, race (White or Black), total energy intake (time-varying average), maximal educational attainment, parental history of CVD (yes or no), pack-years of smoking (time-varying), physical activity level (time-varying average), use of lipid-lowering medications (yes or no), and BMI (time-varying average). ^b^ Time-varying variables that were cumulatively averaged over follow-up at Y0, Y7, and Y20. Y0 predicted events from Y0 to Y7, average of Y0 and Y7 APDQS (average of Y0, Y2, Y5, and Y7 LDL-C) predicted events from after Y7 to Y20, and average of Y0, Y7, and Y20 (average of Y0, Y2, Y5, Y7, Y10, Y15, and Y20 LDL-C) predicted events from after Y20 to Y32. ^c^ 1 SD changes were +0.80 mmol/L for LDL-C, +0.87 mmol/L for Non-HDL-C, +13 for APDQS, and −11 for Keys Score. ^d^ Statistical significance was estimated by modeling APDQS as a continuous variable in the model.

**Table 5:** Multivariable-adjusted HRs (95% CIs) ^a^ of incident CHD and stroke according to quintiles of the time-varying average % of energy from total fat and carbohydrate ^b.^

	Quintile 1	Quintile 2	Quintile 3	Quintile 4	Quintile 5	Per 1 SD Change ^c^	P for Trend ^d^
CHD (Y0-Y32)							
% Energy from total fat							
Range	≤32.3	32.4–35.6	35.7–38.1	38.2–41.0	≥41.1		
Unadjusted cumulative incidence % (n/N)	2.2 (21/940)	2.0 (19/940)	2.3 (22/941)	2.1 (20/940)	3.6 (34/940)		
Adjusted HR	0.79 (0.45–1.40)	0.80 (0.45–1.41)	0.79 (0.46–1.37)	0.67 (0.38–1.16)	1.00 (ref)	0.94 (0.76–1.18)	0.61
% Energy from carbohydrate							
Range	≤41.8	41.9–45.6	45.7–48.7	48.8–52.5	≥52.6		
Unadjusted cumulative incidence % (n/N)	3.1 (29/940)	3.1 (29/940)	2.2 (21/941)	1.5 (14/940)	2.5 (23/940)		
Adjusted HR	0.78 (0.45–1.37)	0.95 (0.55–1.66)	0.68 (0.37–1.26)	0.61 (0.31–1.18)	1.00 (ref)	1.01 (0.82–1.25)	0.91
Both % energy from fat and carbohydrate were mutually adjusted							
% Energy from total fat	0.60 (0.27–1.34)	0.73 (0.36–1.50)	0.81 (0.43–1.50)	0.66 (0.37–1.18)	1.00 (ref)	0.90 (0.64–1.26)	0.53
% Energy from carbohydrate	0.58 (0.26–1.29)	0.77 (0.36–1.63)	0.60 (0.28–1.27)	0.53 (0.25–1.13)	1.00 (ref)	0.94 (0.68–1.29)	0.70
Stroke (Y0-Y32)							
% Energy from total fat							
Range	≤32.3	32.4–35.6	35.7–38.1	38.2–40.9	≥41.0		
Unadjusted cumulative incidence % (n/N)	1.7 (16/940)	1.3 (12/940)	1.9 (18/941)	1.8 (17/940)	1.8 (17/940)		
Adjusted HR	1.60 (0.78–3.27)	1.16 (0.54–2.50)	1.49 (0.75–2.97)	1.35 (0.68–2.69)	1.00 (ref)	1.20 (0.93–1.54)	0.17
% Energy from carbohydrate							
Range	≤41.8	41.9–45.6	45.7–48.7	48.8–52.5	≥52.6		
Unadjusted cumulative incidence % (n/N)	2.0 (19/940)	1.6 (15/940)	1.2 (11/941)	2.1 (20/940)	1.6 (15/940)		
Adjusted HR	0.94 (0.47–1.91)	0.83 (0.40–1.71)	0.67 (0.30–1.47)	1.39 (0.71–2.74)	1.00 (ref)	0.91 (0.71–1.16)	0.45
Both % energy from fat and carbohydrate were mutually adjusted							
% Energy from total fat	1.61 (0.62–4.20)	1.08 (0.43–2.71)	1.44 (0.64–3.24)	1.50 (0.72–3.14)	1.00 (ref)	1.27 (0.88–1.83)	0.21
% Energy from carbohydrate	1.16 (0.45–2.99)	0.94 (0.36–2.44)	0.69 (0.26–1.79)	1.49 (0.67–3.30)	1.00 (ref)	1.08 (0.76–1.52)	0.68

APDQS, A Priori Diet Quality Score; CHD, coronary heart disease; CI, confidence interval; HR, hazard ratio; SD, standard deviation. ^a^ Each row is a separate proportional hazards regression. Model was adjusted for Y0 age, sex, race (White or Black), total energy intake (time-varying average), maximal educational attainment, parental history of CVD (yes or no), pack-years of smoking (time-varying), physical activity level (time-varying average), use of lipid-lowering medications (yes or no), and BMI (time-varying average). ^b^ Time-varying variables that were cumulatively averaged over follow-up at Y0, Y7, and Y20. Y0 predicted events from Y0 to Y7, average of Y0 and Y7 APDQS predicted events from after Y7 to Y20, and average of Y0, Y7, and Y20 predicted events from after Y20 to Y32. ^c^ 1 SD changes were −6 for % energy from total fat and −7.4 for % energy from carbohydrate. ^d^ Statistical significance was estimated by modeling APDQS as a continuous variable in the model.

## Data Availability

The data underlying this article are available in the CARDIA webpage, at https://www.cardia.dopm.uab.edu (accessed on 20 January 2022).

## Supplementary Materials

The following supporting information can be downloaded at: https://www.mdpi.com/article/10.3390/nu14030469/s1. Supplementary Table S1: Multivariable-adjusted HRs (95% CIs) of incident CHD and stroke according to quintiles of the time-varying average APDQS, Keys Score, LDL-C, and non-HDL-C in mutually adjusted models; Supplementary Table S2: Association between change in % energy from total fat and carbohydrate and concurrent changes in LDL-C and Non-HDL-C.
