# Supplementary material for: Simple Nutrient-Based Rules vs. a Nutritionally Rich Plant-Centered Diet in Prediction of Future Coronary Heart Disease and Stroke: Prospective Observational Study in the US
Source: Nutrients. 2022 Jan 21;14(3):469. doi: 10.3390/nu14030469 (PMC8837984; doi:10.3390/nu14030469)
Supplement: Supplementary file 1 [file nutrients-14-00469-s001.zip › nutrients-1553291-SI.pdf]

Supplementary material

Simple nutrient-based rules vs a nutritionally-rich plant-centered diet in prediction of future coronary heart disease and stroke: Prospective Observational Study in the US

Yuni Choi[1], Daniel D. Gallaher[2], Karianne Svendsen[3,4], Katie A. Meyer[5], Lyn M. Steffen[1], Pamela J. Schreiner[1], James M. Shikany[6], Jamal S. Rana[7], Daniel A. Duprez[8], David R. Jacobs Jr[1]

1. Division of Epidemiology and Community Health, University of Minnesota–Twin Cities, Minneapolis, MN, United States
2. Department of Food Science and Nutrition, University of Minnesota–Twin Cities, MN, Saint Paul, United States
3. Department of Nutrition, University of Oslo, Oslo, Norway
4. The Lipid Clinic, Department of Endocrinology, Morbid Obesity and Preventive Medicine, Oslo University Hospital, Oslo, Norway
5. Department of Nutrition and Nutrition Research Institute, University of North Carolina– Chapel Hill, Chapel Hill, NC, United States
6. Division of Preventive Medicine, School of Medicine, University of Alabama at Birmingham, Birmingham, AL, United States
7. Divisions of Cardiology and Research, Kaiser Permanente Northern California, Department of Medicine, University of California, Oakland, CA, United States
8. Cardiovascular Division, Department of Medicine, University of Minnesota–Twin Cities, Minneapolis, MN, United States

Supplementary Tables

Supplementary Table S1. Multivariable-adjusted HRs[a] (95% CIs) of incident CHD and stroke according to quintiles of the time-varying average APDQS, Keys Score, LDL-C, and non-HDL-C[b] in mutually-adjusted models

	Quintile 1	Quintile 2	Quintile 3	Quintile 4	Quintile 5	Per 1 SD change[c]	P for trend[d]
**CHD (Y0-Y32)**							
**Both APDQS and LDL-C were mutually adjusted[e]**							
**LDL-C**							
Unadjusted cumulative incidence % (n/N)	0.5 (5/940)	1.4 (13/942)	2.1 (20/939)	3.2 (30/940)	5.1 (48/940)		
Adjusted HR	1.00 (ref)	2.40 (0.85–6.77)	3.50 (1.31–9.37)	4.89 (1.88–12.7)	6.27 (2.46–15.98)	1.68 (1.40–2.01)	<0.001
**APDQS**							
Unadjusted cumulative incidence % (n/N)	3.6 (34/934)	3.2 (30/950)	2.4 (23/961)	2.2 (20/902)	0.9 (9/954)		
Adjusted HR	1.00 (ref)	0.67 (0.4–1.13)	0.63 (0.36–1.09)	0.63 (0.34–1.17)	0.39 (0.18–0.88)	0.73 (0.56–0.97)	0.03
**Both APDQS and Keys Score were mutually adjusted[e]**							
**Keys Score**							
Unadjusted cumulative incidence % (n/N)	1.7 (16/940)	1.1 (10/940)	3.1 (29/941)	2.7 (25/940)	3.8 (36/940)		
Adjusted HR	0.77 (0.41–1.46)	0.59 (0.31–1.12)	0.99 (0.59–1.66)	0.84 (0.49–1.41)	1.00 (ref)	0.93 (0.73–1.18)	0.53
**APDQS**							
Unadjusted cumulative incidence % (n/N)	3.6 (34/934)	3.2 (30/950)	2.4 (23/961)	2.2 (20/902)	0.9 (9/954)		
Adjusted HR	1.00 (ref)	0.69 (0.41–1.15)	0.71 (0.40–1.24)	0.69 (0.37–1.29)	0.43 (0.19–1.00)	0.75 (0.56–0.99)	0.05
**Both APDQS and Non-HDL-C were mutually adjusted[e]**							
**Non-HDL-C**							
Unadjusted cumulative incidence % (n/N)	0.4 (4/940)	1.1 (10/940)	1.9 (18/941)	3.2 (30/940)	5.7 (54/940)		
Adjusted HR	1.00 (ref)	2.37 (0.74–7.58)	3.57 (1.19–10.7)	6.18 (2.16–17.68)	8.39 (2.97–23.71)	1.72 (1.45–2.06)	<0.001
**APDQS**							

Unadjusted cumulative incidence % (n/N)	3.6 (34/934)	3.2 (30/950)	2.4 (23/961)	2.2 (20/902)	0.9 (9/954)		
Adjusted HR	1.00 (ref)	0.68 (0.41–1.13)	0.63 (0.36–1.11)	0.63 (0.34–1.17)	0.40 (0.18–0.90)	0.73 (0.56–0.97)	0.03
**Stroke (Y0-Y32)**							
**Both APDQS and LDL-C were mutually adjusted**[e]							
**LDL-C**							
Unadjusted cumulative incidence % (n/N)	1.5 (14/940)	1.7 (16/941)	1.4 (13/940)	1.9 (18/940)	2.0 (19/940)		
Adjusted HR	1.00 (ref)	1.45 (0.69–3.06)	1.12 (0.51–2.48)	1.43 (0.68–3.01)	1.29 (0.61–2.74)	1.15 (0.91–1.46)	0.25
**APDQS**							
Unadjusted cumulative incidence % (n/N)	3.5 (33/935)	1.7 (16/948)	1.4 (13/961)	1.2 (11/902)	0.7 (7/955)		
Adjusted HR	1.00 (ref)	0.44 (0.24–0.82)	0.42 (0.21–0.83)	0.61 (0.29–1.27)	0.55 (0.21–1.44)	0.70 (0.50–0.99)	0.043
**Both APDQS and Keys Score were mutually adjusted**[e]							
**Keys Score**							
Unadjusted cumulative incidence % (n/N)	1.3 (12/940)	1.8 (17/940)	1.3 (12/941)	2.1 (20/940)	2.0 (19/940)		
Adjusted HR	0.90 (0.41–1.97)	1.36 (0.70–2.66)	0.63 (0.29–1.37)	1.15 (0.60–2.19)	1.00 (ref)	1.01 (0.75–1.35)	0.97
**APDQS**							
Unadjusted cumulative incidence % (n/N)	3.5 (33/935)	1.7 (16/948)	1.4 (13/961)	1.2 (11/902)	0.7 (7/955)		
Adjusted HR	1.00 (ref)	0.44 (0.23–0.82)	0.41 (0.20–0.83)	0.60 (0.28–1.26)	0.54 (0.20–1.45)	0.70 (0.49–1.00)	0.050
**Both APDQS and Non-HDL-C were mutually adjusted**[e]							
**Non-HDL-C**							
Unadjusted cumulative incidence % (n/N)	1.4 (13/940)	1.5 (14/940)	1.5 (14/941)	1.9 (18/940)	2.2 (21/940)		
Adjusted HR	1.00 (ref)	1.46 (0.67–3.20)	1.27 (0.57–2.81)	1.56 (0.72–3.35)	1.59 (0.74–3.45)	1.23 (0.97–1.57)	0.08
**APDQS**							
Unadjusted cumulative incidence % (n/N)	3.5 (33/935)	1.7 (16/948)	1.4 (13/961)	1.2 (11/902)	0.7 (7/955)		
Adjusted HR	1.00 (ref)	0.44 (0.24–0.82)	0.42 (0.21–0.84)	0.61 (0.29–1.27)	0.55 (0.21–1.44)	0.70 (0.50–0.99)	0.044

APDQS, A Priori Diet Quality Score; CHD, coronary heart disease; CI, confidence interval; HR, hazard ratio; LDL-C, low-density lipoprotein cholesterol; non-HDL-C, non-high-density lipoprotein cholesterol; SD, standard deviation.

[a] Model was adjusted for Y0 age, sex, race (White or Black), total energy intake (time-varying average), maximal educational attainment, parental history of CVD (yes or no), pack-years of smoking (time-varying), physical activity level (time-varying average), use of lipid-lowering medications (yes or no), and BMI (time-varying average).

[b]Time-varying variables that were cumulatively averaged over follow-up at Y0, Y7, and Y20. Y0 predicted events from Y0 to Y7, average of Y0 and Y7 APDQS (average of Y0, Y2, Y5, and Y7 LDL-C) predicted events from after Y7 to Y20, and average of Y0, Y7, and Y20 (average of Y0, Y2, Y5, Y7, Y10, Y15, and Y20 LDL-C) predicted events from after Y20 to Y32.

[c]1 SD changes were +0.80 mmol/L for LDL-C, +0.87 mmol/L for Non-HDL-C, +13 for APDQS, and −11 for Keys Score.

[d] Statistical significance was estimated by modeling APDQS as a continuous variable in the model.

[e]The two predictors were simultaneously included in the model.

Supplementary Table S2. Association between change in % energy from total fat and carbohydrate and concurrent changes in LDL-C and Non-HDL-C[a]

	Adjusted mean change for each 1 SD increment[b]					
	% Energy from total fat change			% Energy from carbohydrate change		
	ß	SE	P-value	*ß*	SE	P-value
**LDL-C, mmol/L**						
7-year change (mean age from 25 y to 32 y), n=3495	−0.013	0.009	0.15	−0.009	0.009	0.35
13-year change (mean age from 32 y to 45 y), n=2360	−0.021	0.009	0.025	0.019	0.010	0.05
20-year change (mean age from 25 y to 45 y), n=2824	−0.019	0.010	0.043	0.011	0.010	0.26
**Non-HDL-C, mmol/L**						
7-year change (mean age from 25 y to 32 y), n=3495	−0.010	0.010	0.31	−0.011	0.010	0.29
13-year change (mean age from 32 y to 45 y), n=2360	−0.012	0.023	0.25	0.007	0.011	0.53
20-year change (mean age from 25 y to 45 y), n=2824	−0.010	0.011	0.36	−0.002	0.011	0.86

APDQS, A Priori Diet Quality Score; LDL-C, low-density lipoprotein cholesterol; non-HDL-C, non-high-density lipoprotein cholesterol.
[a]Each row is a separate linear regression. Model was adjusted for baseline LDL-C (or Non-HDL-C), baseline age, sex, race (White or Black), total energy intake (baseline and change), maximal educational attainment, parental history of CVD (yes or no), pack-years of smoking (baseline and change), physical activity level (baseline and change), use of lipid-lowering medications (yes or no), and BMI (baseline and change).
[b]1 SD changes were −6 for % energy from total fat and −7.4 for % energy from carbohydrate.
